# Effectiveness of transfer learning for enhancing tumor classification with a convolutional neural network on frozen sections

**DOI:** 10.1038/s41598-020-78129-0

**Published:** 2020-12-14

**Authors:** Young-Gon Kim, Sungchul Kim, Cristina Eunbee Cho, In Hye Song, Hee Jin Lee, Soomin Ahn, So Yeon Park, Gyungyub Gong, Namkug Kim

**Affiliations:** 1 Transdisciplinary Department of Medicine & Advanced Technology, Seoul National University Hospital, Seoul, 03080 Korea; 2 Department of Convergence Medicine, Asan Institute of Life Science, University of Ulsan College of Medicine, Asan Medical Center, Seoul, 05505 Korea; 3 Department of Hospital Pathology, Seoul St. Mary’s Hospital, College of Medicine, The Catholic University of Korea, Seoul, 06591 Korea; 4 Department of Pathology, University of Ulsan, College of Medicine, Asan Medical Center, Seoul, 05505 Korea; 5 Department of Pathology, Seoul National University Bundang Hospital, Seoul National University College of Medicine, Gyeonggi, 13620 Korea

**Keywords:** Breast cancer, Biomedical engineering

## Abstract

Fast and accurate confirmation of metastasis on the frozen tissue section of intraoperative sentinel lymph node biopsy is an essential tool for critical surgical decisions. However, accurate diagnosis by pathologists is difficult within the time limitations. Training a robust and accurate deep learning model is also difficult owing to the limited number of frozen datasets with high quality labels. To overcome these issues, we validated the effectiveness of transfer learning from CAMELYON16 to improve performance of the convolutional neural network (CNN)-based classification model on our frozen dataset (N = 297) from Asan Medical Center (AMC). Among the 297 whole slide images (WSIs), 157 and 40 WSIs were used to train deep learning models with different dataset ratios at 2, 4, 8, 20, 40, and 100%. The remaining, i.e., 100 WSIs, were used to validate model performance in terms of patch- and slide-level classification. An additional 228 WSIs from Seoul National University Bundang Hospital (SNUBH) were used as an external validation. Three initial weights, i.e., scratch-based (random initialization), ImageNet-based, and CAMELYON16-based models were used to validate their effectiveness in external validation. In the patch-level classification results on the AMC dataset, CAMELYON16-based models trained with a small dataset (up to 40%, i.e., 62 WSIs) showed a significantly higher area under the curve (AUC) of 0.929 than those of the scratch- and ImageNet-based models at 0.897 and 0.919, respectively, while CAMELYON16-based and ImageNet-based models trained with 100% of the training dataset showed comparable AUCs at 0.944 and 0.943, respectively. For the external validation, CAMELYON16-based models showed higher AUCs than those of the scratch- and ImageNet-based models. Model performance for slide feasibility of the transfer learning to enhance model performance was validated in the case of frozen section datasets with limited numbers.

## Introduction

An advanced machine learning algorithm, i.e., convolutional neural network (CNN)-based deep learning, has recently been adopted to develop models with higher performance than those of conventional algorithms in the field of computer vision tasks^1–3^. The supervised learning-based algorithm, which is a machine learning method for training a function to correctly map an input to an output with corresponding labeling data, generally performs well when sufficient input datasets including labeling data are fed into the CNN-based architecture while training the model. It makes the model robust for predicting an unseen dataset. In the real world, however, it is difficult to obtain not only the dataset but also label the dataset owing to high labeling costs by experts^4,5^. Transfer learning has been used to effectively train the CNN model with the limited dataset, which could enhance the model performance by using an initial model that has been trained with numerous public datasets in different domains, to overcome this issue^6,7^.

As development needs for computer aided-diagnosis (CAD) increased in medical fields such as radiology and pathology, the CNN-based CAD models have been studied and developed. Some studies validated that CNN-based model performance was comparable with experts^8–10^. Deep learning in pathology is becoming increasingly popular. The CNN-based deep learning has been applied to numerous tasks such as detection and segmentation for various organs such as breast, lung, and colon. With regards to detection, mitosis^11–13^ and breast cancer metastasis detection^14,15^ has been studied. For segmentation, colon glands^16^ segmentation for breast cancer metastasis^17^, and multiple cancer subtype regions of lungs^18^ have been studied as well. Breast cancer is the most common cancer in women globally and the treatment is primary tumor surgical removal for patients with localized breast cancer^19^. Fast and accurate diagnosis based on sentinel lymph node (SNL) biopsy with the frozen section^20^, whose quality is inferior to that of the formalin-fixed, paraffin-embedded (FFPE) section, is required; however, this is difficult because of artifacts such as compression, nuclear ice crystals, and nuclear chromatic change artifacts. Moreover, labeling data to train CNN-based deep learning models is also cost- and time-consuming owing to the quality and limited samples.

Transfer learning has been widely used to effectively train models with limited dataset to overcome cost- and time-consuming issue^21^. It enables models to be trained fast and accurately by extracting relatively useful spatial features at the beginning of training learned from large dataset in different domain. In CAMELYON challenge for detection of metastases in SNL biopsy of FFPE, the ImageNet pre-trained model was used as an initialization of training parameters for efficient training^22,23^. In our previous study for metastases classification on frozen section^24^, most participants used ImageNet pre-trained model as an initial parameter as well. By the way, none of study validated effectiveness of CAMLEYON dataset for metastases classification on frozen section as so far.

In this study, we proposed an effective method to train a deep learning-based model with a limited dataset for classification of tumor pathology and the whole slide image (WSI) with the concept of transfer learning. Random initialized pre-trained model and two different pre-trained models based on ImageNet dataset, which is a public dataset for the purpose of classification of 1 k of classes in natural scenes, and the CAMELYON16 dataset, which is a public dataset for tumor slide-based classification of FFPE biopsies in digital pathology were compared. Our contribution is to evaluate and compare training models using scratch learning, CAMLEYON-based pre-trained, and ImageNet-based pre-trained methods with stress tests and various learning configurations.

## Materials and methods

### Data acquisition

This retrospective study was conducted according to the principles of the Declaration of Helsinki and was performed in accordance with current scientific guidelines. The study protocols were approved by the Institutional Review Board Committee of Asan Medical Center (AMC), University of Ulsan College of Medicine, Seoul, Korea and Seoul National University Bundang Hospital (SNUBH), Seoul National University College of Medicine, Gyeonggi, KOREA. The requirements for informed patient consent were waived by the Institutional Review Board Committee of AMC and SNUBH.

All of the sentinel lymph nodes (SLN) in the AMC and the SNUBH were obtained for frozen section routine surgical procedure^24,25^. In the AMC, the WSIs of 297 SLNs were scanned by a digital microscopy scanner (Pannoramic 250 FLASH; 3DHISTECH Ltd., Budapest, Hungary) in MIRAX format (.mrxs). More details of the AMC dataset was introduced in our previous study^24^. In the SNUBH, the WSIs of 228 SLNs were scanned by a digital microscopy scanner (Pannoramic 250 FLASH II; 3DHISTECH Ltd., Budapest, Hungary) in MIRAX format (.mrxs). All WSIs in the AMC were split into 157, 40, and 100 as training, validation, and test sets, respectively. All 228 WSIs was used as an external validation. Demographics for training, validation, and test datasets were described as shown in Table 1. In the case of the CAMELYON16 dataset, only 270 WSIs given for training were split into 216 and 54 WSIs (8:2) for training and validation sets to make a CAMELYON16-based pre-trained model. All training datasets, i.e., 157 WSIs, were split into 2, 4, 8, 20, 40, and 100% at 3, 6, 12, 25, 50, and 157 WSIs. The number of patches were: 12 K, 25 K 52 K 101 K 199 K, and 519 K, respectively.

**Table 1:** Clinicopathologic characteristics of the patients in AMC and SNUBH datasets.

	AMC			SNUBH
	Training set (n = 157)	Validation set (n = 40)	Test set (n = 100)	Test set (n = 228)
Age (median and range)	50 (28–80)	49 (30–68)	47 (34–75)	52 (25–87)
**Sex**				
Female	157 (100%)	40 (100%)	100 (100%)	228 (100%)
**Metastatic carcinoma**				
Present, size > 2 mm	68 (43.3%)	14 (35%)	40 (40%)	84 (36.8%)
Present, size ≤ 2 mm	35 (22.3%)	5 (12.5%)	15 (15%)	44 (19.2%)
Absent	54 (34.4%)	21 (52.5%)	45 (45%)	100 (44%)

AMC, Asan Medical Center; SNUBH, Seoul National University Bundang Hospital.

### Reference standard

All WSIs in the AMC in our dataset were manually segmented by one rater, and the annotations were confirmed by two clinically expert pathologists with 6- and 20-years’ experience in breast pathology. And all WSIs in the SNUBH were manually segmented by one rater, and the annotations were confirmed by an expert breast pathologist with 15-years’ experience. Whole regions of metastatic carcinoma larger than 200 μm in the greatest WSI dimension were annotated. More details of the reference standard are introduced in our previous study^24^.

### Study design

**Figure 1:**
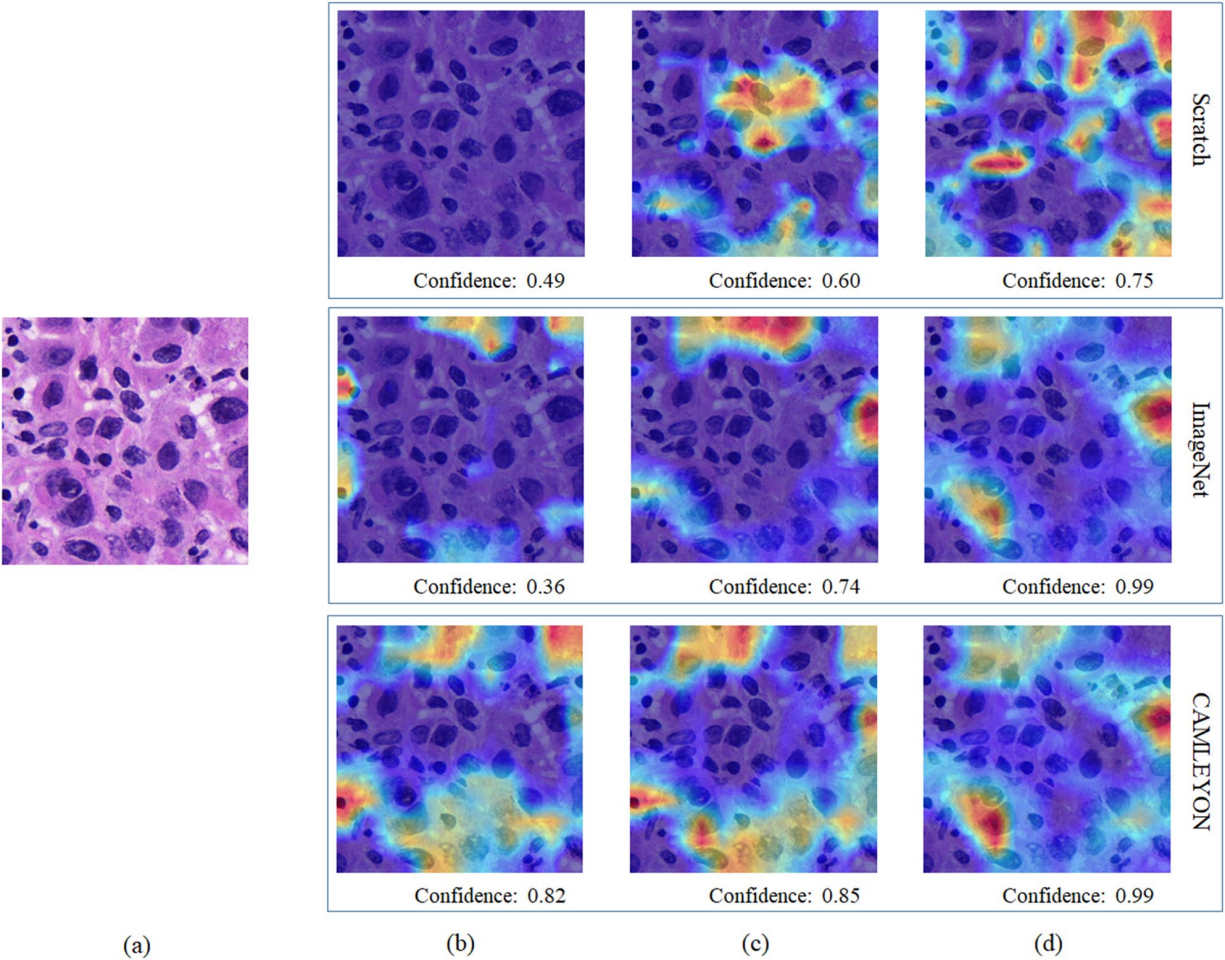
A validation workflow for transfer learning. (**a**) Two initial classification models are trained from two public datasets (i.e., ImageNet and CAMELYON16 datasets). (**b**) Private dataset from AMC is used to train classification models with different initial weights based on scratch- (random initialization), ImageNet- (pre-trained), and CAMELYON16-(pre-trained) based models. Different dataset ratios are selected at 2, 4, 8, 20, 40, and 100% of the total AMC dataset to train models for validation of the transfer learning effectiveness.

In this study, three different model types with initial weights, i.e., (1) scratch-based initial weight that was randomly assigned, (2) ImageNet-based initial weight trained with a public dataset (ImageNet, 1000 classes in natural images), (3) and CAMELYON16-based initial weight trained with another public dataset, (CAMELYON16, 2 classes in H&E pathology images), were used to validate the transfer learning effectiveness as shown in Fig. 1. For more detail, the three different weights were used as initial weights of models trained with different training dataset ratios at 2, 4, 8, 20, 40, and 100% of the training dataset total. Different models with different initial weight and training dataset ratios were evaluated in terms of sensitivity, specificity, accuracy, and area under the curve (AUC) for patch- and slide-level classifications. As an external validation, the SNUBH dataset was used for slide-level classification in AUC terms.

### Patch extraction

Tissue region masks were extracted by combining the H and S color channels after Otsu thresholding to extract tumor and non-tumor patches in WSI^26^. Subsequently, 448 × 448 patch sizes at level zero whose field of view is approximately at 100 µm^2^ were randomly selected within the tissue region and each patch was classified as either tumor or non-tumor patch according to a certain criterion (tumor patch, over 80% of the tumor region; non-tumor patch, none of the tumor region).

The total number of CAMELYON16 patches for tumor and non-tumor patches are 330 K and 88 K for training and validation sets at a 1:1.2 ratio of tumor and non-tumor, respectively. A total number of patches in our datasets for training, validation, and test sets at the same ratio are 519 K, 92 K, and 27 K, respectively.

### CNN training

All models in this study, i.e., CAMELYON16 dataset-based pre-trained model, scratch-based, ImageNet-based, and CAMELYON16-based models, were trained with the same training conditions. Inception v.3^27^ was selected as the classification architecture for validation of transfer learning. It consists of 48 layers to efficiently extract spatial features in different image level from Inception family that enhanced model performance with label smoothing, factorized 7 × 7 convolutional operation. The Inception v.3 showed higher accuracy with lower memory size than other architectures such as VGG^28^ or ResNet^29^ series. The classification models were trained with the same conditions, such as optimizer (SGD, learning rate: 5e−4, momentum: 0.9), loss function (binary-cross entropy), augmentations (zoom range: 0.2, rotation range: 0.5, width and height shift range: 0.1, horizontal and vertical flip), and drop out (0.5). The best model was selected at the lowest validation set loss. Stain normalization was used to make the model robust to different scanning conditions.

Confidence maps were generated by interpolating tumor confidence over all WSI regions. A parameter defining how densely the confidence map is generated (i.e., stride) was 320 pixels, i.e., the resolution of confidence maps is 1/320 of the original WSIs. To reduce noise in the confidence map, we used 3 × 3 Gaussian filtering. The maximum value was selected as the confidence to calculate the receiver operating characteristic curve (ROC).

### Statistical evaluation

For statistical analysis of classification model performance, we performed AUC comparison using Hanley & McNeil method 32 with respect to that of the CAMELYON16-based model for tumor patch and slide classification. Statistical comparisons between AUCs of CAMELYON16-based and others trained models with the same training dataset ratio were performed to determine which the model’s performances with different initial weights were significantly better. Statistical analyses were performed using MedCalc statistical software (MedCalc Software, version 18.2.1, Ostend, Belgium).

## Results

**Figure 2:**
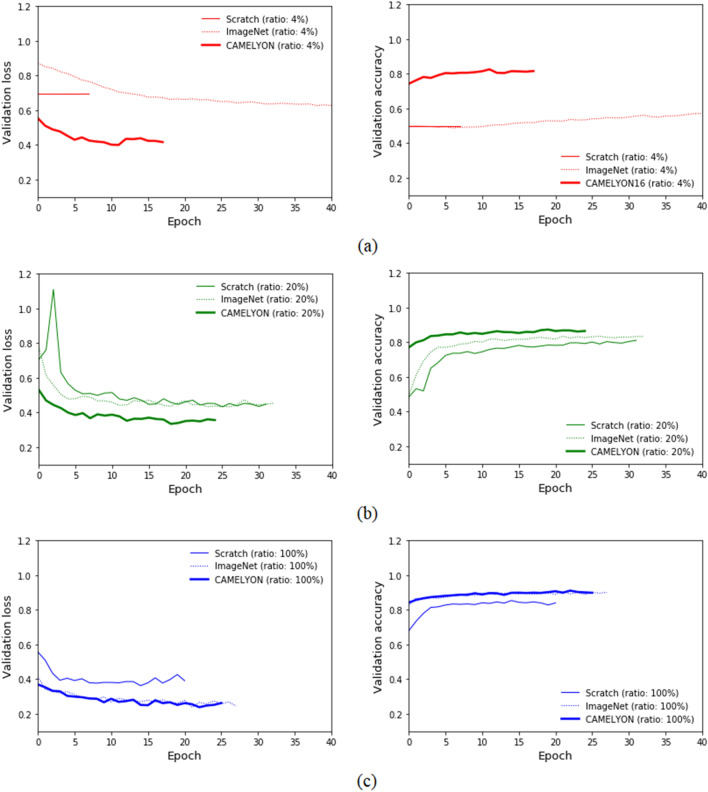
Validation loss and accuracy comparison of scratch-, ImageNet-, and CAMELYON16-based models for different AMC dataset ratios. (**a**) 4%, (**b**) 20%, (**c**) 100% of the total training dataset.

Figure 2 shows plots of training tendency with loss and validation dataset accuracy were observed for three types of models trained with 4, 20, and 100% of the training dataset. Training scratch-based models with 2 to 8% of the training dataset showed no loss and accuracy changes for the validation dataset as shown in Fig. 2a, which indicates that it failed to train the scratch-based models with miniscule training datasets. With 40% or less of the training dataset, CAMELYON16-based models showed not only lower loss and higher accuracy at the first epoch, resulting in fast convergence, but additionally even lower loss and higher accuracy at the last epoch as shown in Fig. 2b. In the case of 100% of the training dataset, ImageNet and the CAMELYON16-based models showed the same tendency for decreased loss and increased accuracy, which showed still lower loss and higher accuracy than that of the scratch-based model as shown in Fig. 2c.

Performance comparisons of patch- and slide-level classifications of whether each patch or slide contains a tumor or not are listed in Table 2. In the patch-level performance case on the AMC dataset, CAMELYON16-based models trained with all training dataset ratios demonstrated significantly higher AUCs at 0.843, 0.881, 0.895, 0.912, 0.929, and 0.944 than those of scratch- and ImageNet-based models except for the ImageNet-based model trained with 100% of the training dataset. In the slide-level performance case on the AMC dataset, CAMELYON16-based models trained with all training dataset ratios showed significantly higher AUCs at 0.814, 0.874, 0.873, 0.867, 0.878, and 0.886 than those of scratch- and ImageNet-based models except for that of the ImageNet-based model trained with 40 and 100% of the training dataset.

**Table 2:** Performance comparison of models based on different initial weights for different ratios of the training dataset.

Ratio	AMC					SNUBH
	Patch-level				Slide-level	Slide-level
	Sensitivity	Specificity	Accuracy	AUC	AUC	AUC
**Scratch-based model**						
2%	–	–	–	–	–	–
4%	–	–	–	–	–	–
8%	–	–	–	–	–	–
20%	0.737	0.848	0.798	0.887**	0.689**	0.540**
40%	0.715	0.873	0.801	0.897**	0.703**	0.495**
100%	0.627	0.930	0.791	0.914**	0.781**	0.437**
**ImageNet-based model**						
2%	0.060	0.989	0.5669	0.825**	0.739**	0.592*
4%	0.284	0.976	0.660	0.869**	0.724**	0.625
8%	0.184	0.995	0.626	0.885**	0.794**	0.667
20%	0.431	0.966	0.722	0.894**	0.847*	0.723
40%	0.483	0.972	0.749	0.919**	0.870	0.819**
100%	0.660	0.968	0.828	0.943	0.888	0.798
**CAMELYON16-based model**						
2%	0.475	0.898	0.705	0.843	0.814	0.689
4%	0.617	0.907	0.775	0.881	0.874	0.667
8%	0.474	0.967	0.742	0.895	0.873	0.695
20%	0.494	0.973	0.755	0.912	0.867	0.763
40%	0.534	0.972	0.772	0.929	0.878	0.749
100%	0.656	0.969	0.827	0.944	0.886	0.804

In the AMC dataset, sensitivity, specificity, and accuracy at threshold 0.5, and AUC were measured for patch-level evaluation and AUC for slide-level evaluation was measured on AMC and SNUBH datasets.

Statistical comparisons between AUCs of CAMELYON16-based model and others trained with the same ratio of the training dataset were performed to determine whether the model’s performance with different initial weights were significant.

AMC, Asan Medical Center; SNUBH, Seoul National University Bundang Hospital.

*p-value < 0.05, **p-value < 0.0005.

In the external validation dataset with SNUBH, the AUCs are measured for ImageNet-based and CAMELYON16-based model trained with 2, 4, 8, 20, 40, and 100% of the training set were 0.592, 0.625, 0.667, 0.723, 0.819, and 0.798; and 0.689, 0.667, 0.695, 0.763, 0.749, and 0.804, respectively. In the scratch-based model case, the model performance was observed at between 0.437 and 0.540. The CAMELYON16-based model trained with all ratios of the total training set showed higher AUCs than those of the ImageNet-based model except for 40% of the total training dataset.

**Figure 3:**
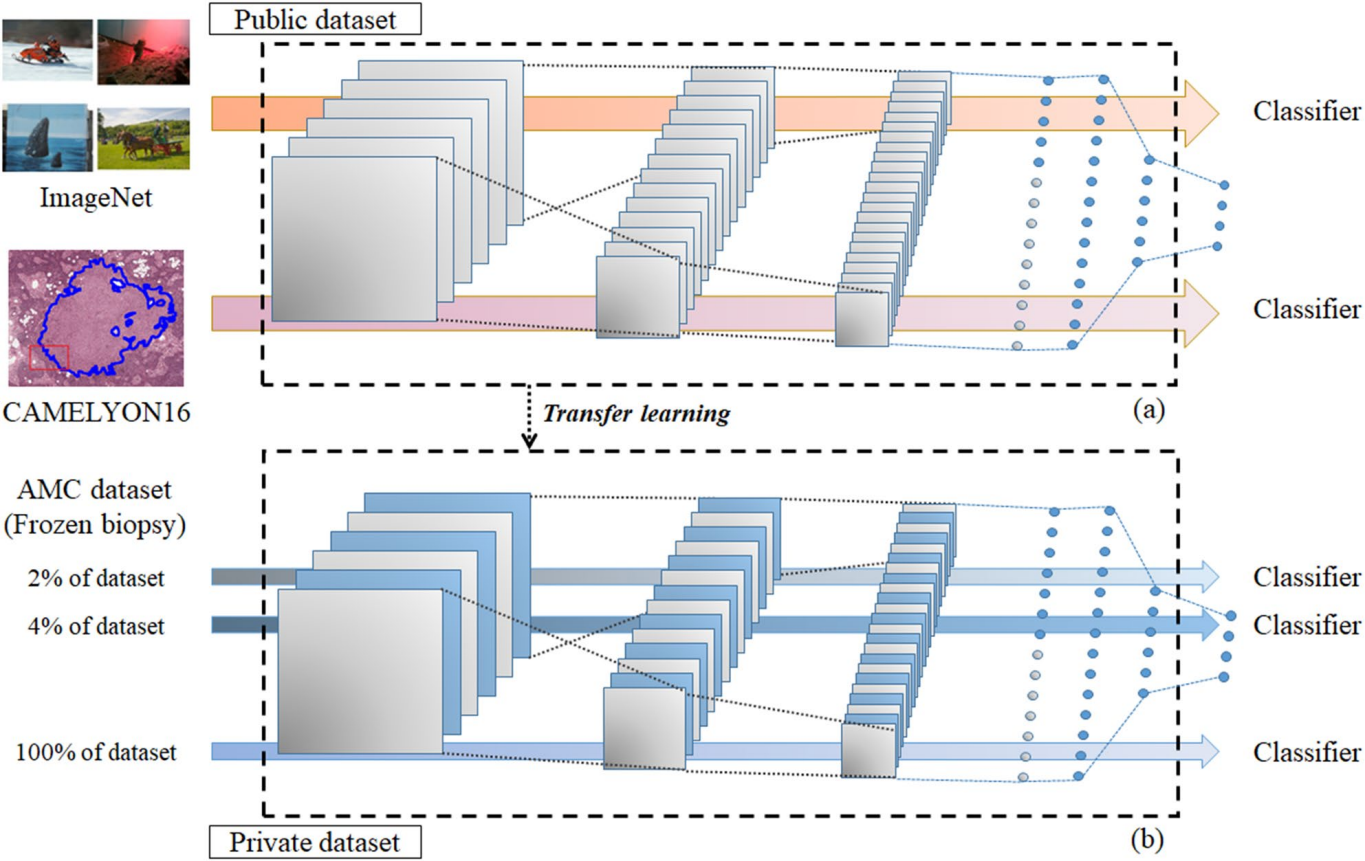
An example of Grad-CAMs for tumor patch with scratch-, ImageNet-, and CAMELYON16-based models trained with different dataset ratios. Confidence below each result indicates the value of the last fully connected layer in each model. (**a**) Input patch, Grad-CAM results of models trained with (**b**) 4%, (**c**) 20%, and (**d**) 100% of the total AMC dataset.

Figure 3 shows an example of Grad-CAMs for tumor patch with scratch-, ImageNet-, and CAMELYON16-based models trained with different dataset ratios. Confidence denotes an output value of the last fully connected layer in each model. All models trained with 20 and 100% of the training dataset correctly predicted an input patch as a tumor patch while in the case of using 4% of the training dataset, only a CAMELYON16-based model correctly predicted with higher confidence of 0.82. In the same condition, scratch- and ImageNet-based models incorrectly predicted the input patch as a normal patch with low confidence levels of 0.49 and 0.36, respectively.

## Discussion

We validated transfer learning using ImageNet and CAMELYON16 to reduce intensive labor costs by comparing with the scratch-based model. Three model types based on scratch, ImageNet, and CAMELYON16 dataset for different ratios such as 2, 4, 8, 20, 40, and 100% of the training dataset were trained and evaluated.

The scratch-based models failed to train with ratios less than or equal to 8% of the training dataset due to the very limited dataset as shown in Fig. 2a. The scratch-based models, however, began to achieve training as the training dataset increases as shown in Table 2. CAMELYON16-based models trained with all ratios of the training dataset were trained with lower losses and higher accuracies for the validation dataset than those of scratch-based models at the first and last epochs. Its tendency resulted in significantly higher AUCs for patch and slide classification with tumors, which could also save time in effective model training.

In the case of comparisons between ImageNet- and CAMELYON16-based models, the CAMELYON16-based models showed significantly higher AUCs for patch- and slide-level classification when provided with equal or less than 40% and 20%, respectively. Loss and accuracy for both models trained with 100% were close to each other at every epoch, resulting in comparable AUCs for patch-level classification at 0.943 and 0.944 and slide-level classification at 0.888 and 0.886.

External validation with SNUBH was conducted to validate the transfer learning effectiveness. Though overall AUCs of ImageNet-based and CAMELYON16-based models for all training dataset ratios were slightly lower than that in the AMC dataset, a tendency showing increasing AUCs was observed as additional datasets were used for model training. The primary difference between AMC and SNUBH is MPP (micro-meter per pixel) that is related to the definite size for each pixel. This parameter is determined when scanning the whole slide. The MPP of AMC and SNUBH datasets are 0.24 and 0.50, respectively. Considering patches at the same level, the patch resolution in the SNUBH dataset is approximately half that of the AMC dataset patches. A harsher resizing augmentation during training was used to reduce variances between the AMC and SNUBH datasets to overcome this issue.

Grad-CAM of three model types trained at 4, 20, and 100% of the training dataset was used by showing a heat-map as shown in Fig. 3 to observe how reasonably each model was trained. Grad-CAMs of all model types showed higher confidence for tumor patches as the training datasets increase. However, the CAMELYON16-based model trained with 4% of the training dataset showed higher tumor patch confidence at 0.82 than that of scratch- and ImageNet-based models at 0.49 and 0.36. Scratch-based models trained with equal or less than 8% of the training dataset predicted all patches as non-tumor patches at 0.51 confidence, which indicates overfitting for the non-tumor class.

Additional experiments with various ranges of learning rates that are considered as a major factor affecting model performance was conducted by setting the value from 5e−2 (0.05) to 5e−6 (0.000005) to see a tendency of model performance in terms of AUC for patch-level evaluation in AMC dataset. Model performances are listed as shown in Table 3. In the result, a range of learning rate from 5e−4 to 5e−5 was good enough to efficiently train all types of models including scratch-based model. When we set the learning rate greater than or equal to 5e−3, the most training was divergent at the beginning of training. As the learning rates were set to less than 5e−5, the model trainings needed more epochs for convergence and those were converged at relatively higher loss level, which resulted in decreasing model performance.

**Table 3:** Performance comparison of models based on different initial weights and learning rates for different ratios of the training dataset.

Ratio	Learning rate	AUC		
		Scratch-based model	ImageNet-based model	CAMELYON-based model
20%	5e−2	0.516	0.511	0.572
	5e−3	0.623	0.766	0.799
	5e−4	0.887	0.894	0.912
	5e−5	0.890	0.891	0.920
	5e−6	0.813	0.872	0.910
40%	5e−2	0.546	0.599	0.597
	5e−3	0.759	0.803	0.812
	5e−4	0.897	0.919	0.929
	5e−5	0.881	0.905	0.930
	5e−6	0.822	0.824	0.931
100%	5e−2	0.546	0.616	0.631
	5e−3	0.754	0.804	0.811
	5e−4	0.914	0.943	0.944
	5e−5	0.921	0.944	0.938
	5e−6	0.911	0.906	0.901

AUC was measured for patch-level evaluation in AMC dataset.

Our study has several limitations. First, the tumor patch was selected to train models when the tumor region is more than 80% of each patch, which resulted in specific models. Patch classification performance depends on selection criteria and different criteria may slightly affect the model’s characteristics. Second, a method of slide-level classification using the maximum value of the confidence map seems so straightforward that the noise in the confidence map could significantly affect the results. Its strategy should be addressed with a robust method such as a random forest classifier with local and global features.

## Conclusions

Training of a CNN model with a limited number of pathology datasets is an essential task to reduce intensive labor costs. To overcome this issue on intraoperative frozen SNL biopsy tissue, we validated the effectiveness of transfer learning with pre-trained models of open datasets such as the ImageNet and CAMELYON16 datasets obtained from FFPE tissue by comparing them with a scratch-based model. In our results, the CAMELYON16-based models trained with all training dataset ratios for patch and slide classification that have tumors showed significantly higher AUCs than those of the scratch-based models. When using the 40% or less (i.e., 62 WSIs) of the training set, the CAMELYON16-based models showed higher AUCs than those of ImageNet-based models for patch classification. With the limited datasets, transfer learning using the FFPE tissue-based dataset, could enhance model performance for tumor classification in the intraoperative frozen tissue-based dataset.
